# Miss Charlotte Louisa Ellaby

**Published:** 1909-05-22

**Authors:** 


Miss Charlotte Louisa Ellaby, M.D.(Paris), L.S.A., died on May 13 at the age of 55. She obtained the M.D. degree of the University of Paris in 1884, and became a licentiate of the Society of Apothecaries in 1889. She had been second physician to the Cama Hospital in Bombay, and was ophthalmic surgeon and subsequently consulting ophthalmic surgeon to the New Hospital for Women. Dr. Ellaby was lecturer on ophthalmic surgery at the London School of Medicine for Women and a member of the Faculty of Medicine of London University, and has contributed several papers in French to the *Archives d'Ophthalmologie.*

